# Label-free drug response evaluation of human derived tumor spheroids using three-dimensional dynamic optical coherence tomography

**DOI:** 10.1038/s41598-023-41846-3

**Published:** 2023-09-16

**Authors:** Ibrahim Abd El-Sadek, Larina Tzu-Wei Shen, Tomoko Mori, Shuichi Makita, Pradipta Mukherjee, Antonia Lichtenegger, Satoshi Matsusaka, Yoshiaki Yasuno

**Affiliations:** 1 Computational Optics Group, University of Tsukuba, Tsukuba, Ibaraki 305-8573 Japan; 2 Department of Physics, Faculty of Science, Damietta University, New Damietta City, Damietta 34517 Egypt; 3 Clinical Research and Regional Innovation, Faculty of Medicine, University of Tsukuba, Ibaraki, 305-8575 Japan; 4 Center for Medical Physics and Biomedical Engineering, Medical University of Vienna, Währinger Gürtel 18-20, 4L, 1090 Vienna, Austria

**Keywords:** Interference microscopy, Cancer, Breast cancer, Cancer imaging, Drug screening, Imaging and sensing

## Abstract

This study aims at demonstrating label-free drug-response-patterns assessment of different tumor spheroids and drug types by dynamic optical coherence tomography (D-OCT). The study involved human breast cancer (MCF-7) and colon cancer (HT-29) spheroids. The MCF-7 and HT-29 spheroids were treated with paclitaxel (Taxol; PTX) and the active metabolite of irinotecan SN-38, respectively. The drugs were applied with 0 (control), 0.1, 1, and 10 μM concentrations and the treatment durations were 1, 3, and 6 days. A swept-source OCT microscope equipped with a repeated raster scanning protocol was used to scan the spheroids. Logarithmic intensity variance (LIV) and late OCT correlation decay speed (OCDS$_l$) algorithms were used to visualize the tumor spheroid dynamics. LIV and OCDS$_l$ images visualized different response patterns of the two types of spheroids. In addition, spheroid morphology, LIV, and OCDS$_l$ quantification showed different time-courses among the spheroid and drug types. These results may indicate different action mechanisms of the drugs. The results showed the feasibility of D-OCT for the evaluation of drug response patterns of different cell spheroids and drug types and suggest that D-OCT can perform anti-cancer drug testing.

## Introduction

Cancer is one of the deadliest diseases worldwide^1,2^. The cancer mortality rate could be reduced through early detection and appropriate selection of anti-cancer drugs. To properly select the anti-cancer drugs, patient-derived tumor cells can be cultivated as three-dimensional (3D) cell culture, so-called tumor spheroid. Tumor spheroid closely mimics the structural organization, the gradients of oxygen and nutrients, growth kinetics, and metabolic rates of in vivo solid tumors^3–6^. In addition, it mimics the response or resistance of in vivo tumors to chemotherapy and radiotherapy^7,8^. Tumor spheroids have therefore been used widely in the field of anti-cancer drug investigations. Where an anti-cancer drug’s efficacy can be evaluated based on its impact on the spheroid’s morphology and cell viability^9–12^.

Spheroid drug response evaluation has been widely performed using conventional methods, e.g., staining histology^13,14^, fluorescence microscopy^15–17^, and bright field microscopy^18,19^. However, most of these methods are invasive (use chemical labeling and/or tissue slicing), 2D imaging modalities, and have a limited penetration depth of a few hundred microns. These limitations made the standard methods not suitable for label-free, entire-depth, and volumetric evaluation of spheroids.

Optical coherence tomography (OCT)^20^ and its microscopic version so-called optical coherence microscopy (OCM)^21^ are biomedical imaging modalities that provide high-resolution, label-free, non-invasive, and 3D imaging over a depth of a few millimeters. There were some demonstrations of tumor spheroids imaging using OCT. OCT signal attenuation coefficient was used to highlight the necrotic regions in tumor spheroids^22,23^. Volumetric growth of tumor spheroids was quantified using standard OCT imaging^24^. In addition, the regional differences in tumor spheroid drug response have been evaluated using OCT-based cell density quantification^25^.

Dynamic OCT (D-OCT) is an emerging method that provides tissue activity/intra-cellular motion contrasts of fresh ex vivo and in-vitro samples^26–32^. D-OCT represents a combination of high-speed OCT systems with signal processing of sequentially acquired OCT frames. D-OCT has been implemented by combining time domain full-field OCT and statistical and/or time-frequency analysis of the sequentially captured OCT frames^26,27^. It enabled the *en face* imaging of the cell metabolism in fresh excised mouse organs. Recently, 3D dynamic full field OCT of retinal organoids^28^ and retinal pigment epithelium stress models^32^ were demonstrated. D-OCT has also been implemented with scanning type OCT devices. It enabled both the cross-sectional intracellular activity imaging of several ex vivo^30^ and in-vitro samples including tumor spheroid^31^. Volumetric D-OCT has been also implemented with scanning type OCT^29,33–37^. However, it requires either an ultra-fast OCT system^29,33,34^ or long volumetric acquisition time^35–37^. By combining a standard-speed swept-source OCT device with a custom-designed 3D scanning protocol, we recently demonstrated 3D D-OCT imaging of tumor spheroids in 52.4 s^38,39^. To visualize the spheroid intracellular activity, we developed two algorithms: logarithmic intensity variance (LIV) and late OCT correlation decay speed (OCDS$_l$). LIV is a measure of the magnitude of the logarithmic (dB-scaled) OCT time-sequential signal fluctuations. On the other hand, OCDS$_l$ quantifies the rate of the decorrelation of OCT time-sequence at a correlation time range of [204.8 ms,1228.8 ms]. The LIV was believed to indicate the magnitude of the intracellular motility, while OCDS$_l$ is specifically sensitive to slow intracellular motility^39^. More details about the algorithms can be found at the Methods section.

D-OCT was shown to visualize the cellular response of human breast (MCF-7 cell-line) tumor spheroid to paclitaxel (Taxol; PTX)^39^. PTX affects the tumor cells by stabilizing the microtubules^40,41^. It stops the tumor cell mitosis and corrupts the cell structures, which are maintained by the microtubules^42,43^. There are several drugs other than PTX can be used to treat breast cancer, including tamoxifen citrate (TAM), doxorubicin (DOX), and so on. The mechanism of drug interaction with tumor cells is different among these drug types. For example, TAM inhibits the estrogen receptors’ transcription. In addition, it up-regulates the production of transforming growth factor $\beta$ (TGF- $\beta$ ) and down-regulates the insulin-like growth factor 1 (IGF-1) and protein kinase C (PKC)^44,45^. It induces cell apoptosis. DOX inhibits the growth of tumor cells by blocking the topoisomerase II, damages the cell’s DNA and membrane, and induces cell apoptosis^46,47^. On the other hand, other types of anti-cancer drugs can be used to treat other types of cancer. For example, an active metabolite of irinotecan (SN-38) can be used for colon cancer treatment^48,49^. It breaks the DNA by inhibiting the topoisomerase I (Topo-I) enzyme and leads to cell apoptosis^48,50^. As each drug has a different action mechanism with the tumor cells, it might lead to different morphological and tissue activity alterations of tumor spheroids. In order to establish the D-OCT as one of the standard tools for drug-effect assessment, it is important to validate that D-OCT can visualize and quantitatively assess the different drug interactions with different tumor cell types.

In this study, we are going to show that 3D D-OCT can visualize specific drug-response patterns of human breast cancer (MCF-7) and colon cancer (HT-29) spheroids treated with PTX and SN-38, respectively. The study involved three concentrations of each drug, e.g., 0.1, 1, and 10-μM in addition to control cases. The drugs were applied for one, three, and six days. Image-based 3D observation of the time-course drug response shows different spatial patterns of the drug effects among the different tumor and drug types. In addition, quantification of the D-OCT and the spheroid morphology reveals different time courses among the cell and drug types.

## Results

In this study, we used a custom-built 1.3-μm swept-source OCT device^51^ with a repeated raster scanning protocol^39^. At each location in the tissue, 32 frames were captured with a frame repeating time of 204.8 ms. The volumetric tomography is captured in 52.4 s as explained in detail in the Methods section. To visualize the microscopic motility of the tumor spheroid, the temporal fluctuations of the sequentially captured dB-scaled OCT signal are analyzed using two types of D-OCT algorithms; logarithmic intensity variance (LIV) and OCT correlation decay speed (OCDS$_l$). LIV and OCDS$_l$ are supposed to be sensitive for the magnitude and speed of intracellular motility, respectively^31,39^. Fluorescence microscopy with living and dead cell markers [calcein-AM (green) and propidium iodide (red)] is performed as a reference.

### MCF-7 spheroid response to PTX

**Figure 1:**
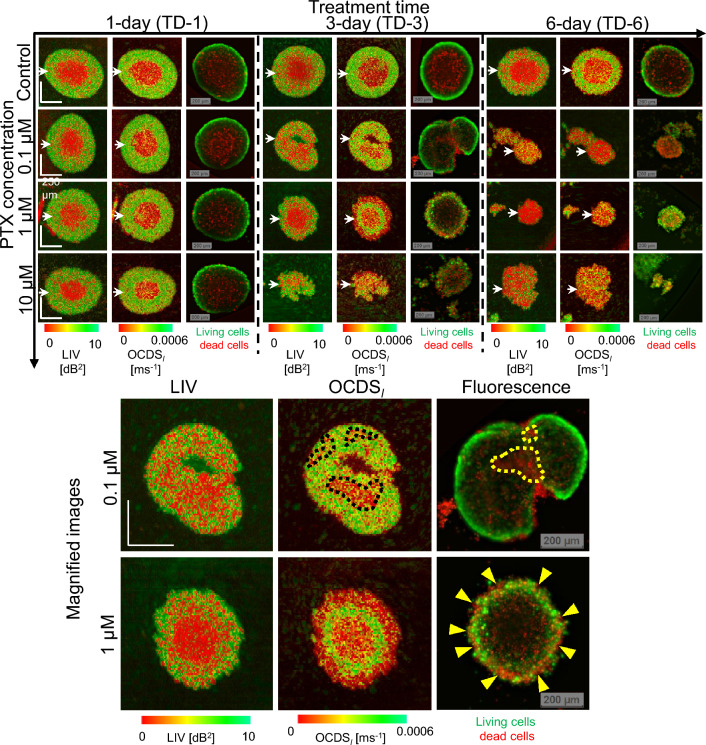
LIV, OCDS$_l$, and fluorescence images of MCF-7 spheroid treated with PTX. The images are *en face* slices at around the center of the spheroids. The B-scan cross-sections and the intensity OCT images are available in the Supplementary materials (as Fig. S2 and Fig. S1, respectively.) Although the difference between the control sample and the PTX-treated samples is not obvious with the 1-day treatment, the 3- and 6-day-treated spheroids showed significantly different appearances to the control sample. The appearances of the fluorescence images are almost consistent with those of the LIV and OCDS$_l$ images. The upper row of magnified images shows spheroid treated with 0.1-μM PTX, while the lower row shows one treated with 1-${\upmu }$M PTX. The treatment time in both cases is 3 days. The appearance of the fluorescence images is more consistent with the OCDS$_l$ images than LIV images. The scale bars represent 200 μm.

Figure 1 summarizes the *en face* LIV, OCDS$_l$, and fluorescence images of the MCF-7 spheroids treated with PTX. In the control case (the first row), the low LIV and low OCDS$_l$ (red) regions increase over time. These low-dynamics regions at the spheroid center may correspond to the dead cells (red) shown in the fluorescence images. It is also found that the boundaries between the high and low signal regions are clearer in the OCDS$_l$ images than in the LIV images.

On the first day of treatment (treatment-day-1; TD-1), the PTX-treated spheroids show similar appearances to the control sample. However, as the treatment time increases, the spheroids show shape corruption, volume reduction, and decrease of the LIV and OCDS$_l$ signals. The fluorescence images show an increase of dead cells over time in addition to shape corruption.

Notably, the LIV and OCDS$_l$ images of the 0.1-μM and 1-μM cases on TD-3 show completely different patterns (as shown in the magnified images). In the 0.1-μM case, tessellated low dynamics (red) patterns appear in the OCDS$_l$ image (black dotted shapes), while the LIV image shows low dynamics (red) overall. The fluorescence image also shows some domains composed of dead cells (red, yellow dashed lines). In the 1-μM case, the LIV image shows two domains including a central low-dynamics (red) region and a peripheral region that is a mixture of low (red) and high (green) dynamics. In contrast, the OCDS$_l$ image shows three concentric domains of low, high, and low dynamics. The fluorescence image shows dead cells (red) at the periphery (indicated by yellow arrowheads) and the central regions, with live cells (green) located in between. In both cases, the appearances of the fluorescence images are more consistent with OCDS$_l$ than LIV images.

For the reference of the readers, OCT intensity images corresponding to the images shown in Fig. 1 are presented in the supplementary material (Fig. S1). The fast-scan cross-sections of LIV and OCDS$_l$ extracted at the locations indicated by the white arrowheads in Fig. 1 are also presented in a supplementary figure (Fig. S2).

Images of additional spheroids measured under each treatment condition are presented in a supplementary figure (Fig. S3) and are consistent with the images in Fig. 1.

**Figure 2:**
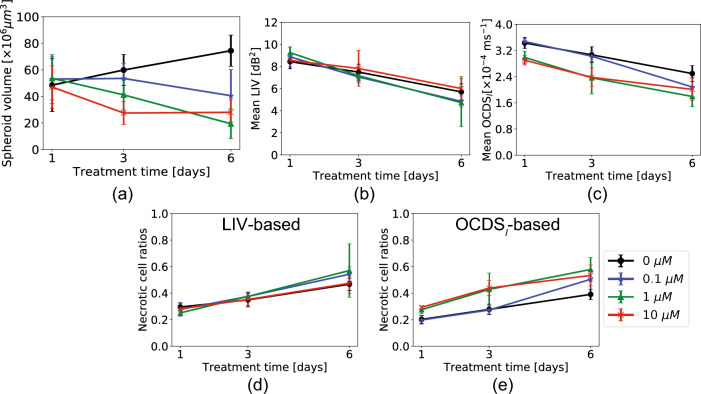
Quantitative analysis of the MCF-7 spheroids treated with several concentrations of Taxol. (**a**) Spheroid volume, (**b**) mean LIV, (**c**) mean OCDS$_l$, and (**d**) and (**e**) necrotic cell ratios computed based on LIV and OCDS$_l$, respectively. The plotted values represent the averages of five samples in each case, while the error bars represent the ± standard-deviation range.

In Fig. 2, the spheroid volume, the mean LIV, the mean OCDS$_l$, the LIV-based necrotic cell ratio, and the OCDS$_l$-based necrotic cell ratio are plotted as functions of the treatment time. Although the *en face* images of the control spheroid presented in Fig. 1 don’t show a clear change in spheroid size over time, the spheroid volume is increasing over time as shown in Fig. 2a. And this increase may be due to the growth of the spheroids. The spheroids treated with 0.1-μM and 1-μM of PTX, on the other hand, show decreasing volume trends over time. In the case of 10-${\upmu }$M, the spheroid volume reduced from TD-1 to TD-3 and stabilized from TD-3 to TD-6. The following considerations could explain the different spheroid volume trends at low and high concentrations of PTX. The influence of 10-μM PTX on the spheroid’s volume is suspected to be faster than that of lower concentrations (0.1-μM and 1-μM). As a result, the spheroid volume reduction is saturated at TD-3 of 10-μM and no more reduction of the spheroid volume is observed. On the other hand, the 0.1-${\upmu }$M and 1-μM of PTX may gradually and slowly affect the spheroid volume and continue to reduce it until TD-6.

The mean LIV and mean OCDS$_l$ over the entire spheroid volume (Fig. 2b, c) show decreasing trends over time for all PTX concentrations. The control spheroid also shows decreasing trends of mean LIV and mean OCDS$_l$ over the incubation time. We believe that this decrease of the mean LIV and mean OCDS$_l$ is accounted by the enlargement of the necrotic core area caused by shortage of oxygen and nutrients at the spheroid center, as shown in Fig. 1. A significant reduction in the mean LIV was found for all PTX concentrations, except for 10-μM from TD-1 to TD-3 (*P* = 0.018 and 0.006 for the 0.1 and 1.0-μM) and from TD-3 to TD-6 (*P* = 0.006 and 0.018 for the 0.1 and 1.0-${\upmu }$M). For the 10-μM samples, the TD-1-to-TD-3 and TD-3-to-TD-6 reductions of mean LIV were insignificant (*P* = 0.071 for both cases), but TD-1-to-TD-6 reduction was significant (*P* = 0.006). For the control spheroids, the reduction of mean LIV was significant from TD-1 to TD-3 and from TD-3 to TD-6 (*P* = 0.047 and 0.006, respectively). For the mean OCDS$_l$, significant reductions were also found for two concentrations of PTX from TD-1 to TD-3 (*P* = 0.006 and 0.018 for the 0.1 and 1-μM, respectively) and from TD-3 to TD-6 (*P* = 0.006 and 0.030 for 0.1 and 1-μM, respectively). For the 10-μM case, the reduction from TD-1 to TD-3 (*P* = 0.006) was significant, although it was not significant from TD-3 to TD-6 (*P* = 0.148). This may occur because the mean OCDS$_l$ has already become very low on TD-3. For the control spheroids, the mean OCDS$_l$ reduction was significant from TD-1 to TD-3 and from TD-3 to TD-6 (*P* = 0.030 and 0.006, respectively). All the *P*-values of these tests are summarized in Table S1 (supplementary).

The necrotic cell ratios obtained with both LIV and OCDS$_l$ (Fig. 2d,e, respectively) increase over the treatment times for all PTX concentrations. An increase in the necrotic cell ratios in the control case might indicate the increase of necrotic core area over the incubation time. Significant increases in the LIV-based necrotic cell ratios were found from TD-1 to TD-3 (*P* = 0.030, 0.006, and 0.047 for 0.1-μM, 1-μM and 10-${\upmu }$M, respectively), and from TD-3 to TD-6 (*P* = 0.006, 0.047, and 0.030 for 0.1-μM, 1-${\upmu }$M, and 10-μM, respectively) for all PTX concentrations. For the control sample, the observed increase was significant from TD-3 to TD-6 (*P* = 0.006). A significant increase in the necrotic cell ratio was also found in the OCDS$_l$-based necrotic cell ratio from TD-1 to TD-3 for all PTX concentrations(*P* = 0.018, 0.010, and 0.006 for the 0.1-μM, 1-μM, and 10-μM). Similar increases were also found from TD-3 to TD-6 for all concentrations, except for the 10-μM case (*P* = 0.006 and 0.030 for the 0.1-μM and 1-μM, respectively). For the control spheroids, the increase of OCDSμ-based necrotic cell ratio was significant from TD-1 to TD-3 and from TD-3 to TD-6 (*P* = 0.030 and 0.006, respectively). All statistics are summarized in Table S1 (supplementary).

### HT-29 spheroid response to SN-38

**Figure 3:**
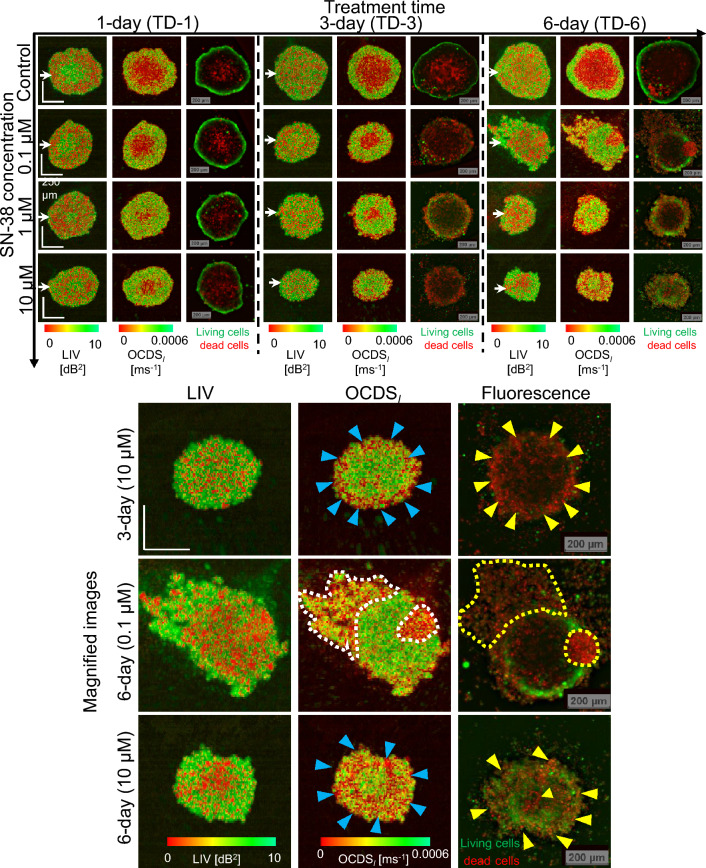
*En face* LIV, OCDS$_l$, and fluorescence images of an HT-29 spheroid treated with SN-38. The LIV and OCDS$_l$ patterns are very different to those for MCF-7 when treated with PTX (Fig. 1). Specifically, the LIV images of the TD-1 spheroids show high LIV (green) at the center surrounded by low LIV (red). After longer treatment times, the LIV images show a granular appearance. The OCDS$_l$ images show concentric domain structures. In addition, the OCDS$_l$ and fluorescence images are found to be highly correlated. Magnified images of HT-29 spheroids with sever volume reduction and shape corruption. High levels of pattern correlation between the OCDS$_l$ and fluorescence patterns are observed. The scale bars represent 200 μm.

Figure 3 summarizes the *en face* LIV, OCDS$_l$, and fluorescence images of the HT-29 spheroids after treatment with SN-38. For the TD-1 spheroids at all the SN-38 concentrations, including the (control) case, the LIV appearance is contrary to that of the MCF-7 spheroids with PTX. Specifically, the center region shows high LIV (green), and this area is surrounded by low LIV (red) regions. For the 10-μM case, in particular, the low LIV region is further surrounded by a moderately high LIV periphery. In contrast, the appearance of the OCDS$_l$ images is similar to that in the MCF-7 case. Namely, the center shows low OCDS$_l$, and this area is surrounded by a high OCDS$_l$ periphery.

For the longer treatment times (TD-3 and TD-6), the LIV images show granular patterns of high and low LIV, and clear domain structures are not observed. This appearance is contrary to that of the MCF-7, and is cell-type dependent. In contrast, most of the OCDS$_l$ images show double or triple concentric domain structures. These structures may be the result of two factors, which include drug effect from the peripheral side and hypoxia or lack of nutrients starting from the center region. The appearances of the OCDS$_l$ images are quite consistent with the fluorescence images. The D-OCT and fluorescence images show clear volume reductions and/or structural corruption under the SN-38 treatment.

The magnified images of representative spheroids show both volume reduction and structural corruption. These images demonstrate the high pattern correlation between the OCDS$_l$ and fluorescence images, as indicated by the arrowheads and dashed lines. In contrast, the LIV patterns are not similar to the OCDS$_l$ and fluorescence images.

**Figure 4:**
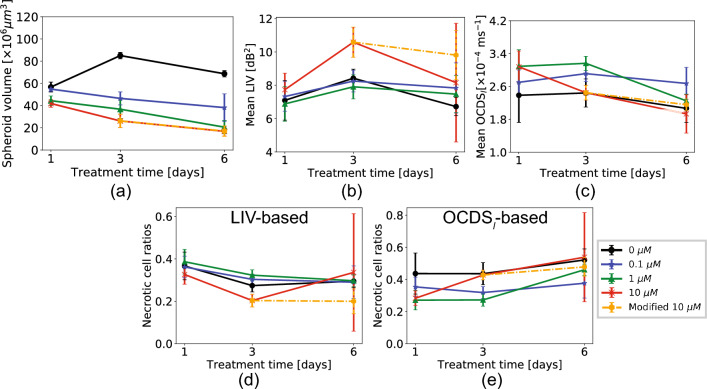
Morphological and dynamics analysis of the HT-29 spheroid when treated with SN-38. The plots are presented in the same order as those in Fig. 2. The plotted values represent the average values of five samples (N=5) under each treatment condition and the error bars represent the ± standard-deviation range.

**Figure 5:**
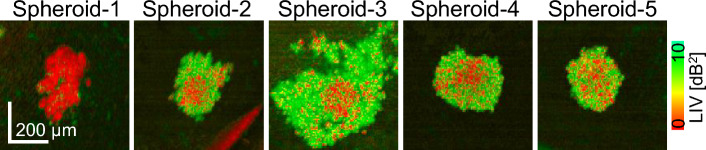
LIV images of the five individual spheroids when treated with 10 μM of SN-38 for 6 days. The first spheroid showed exceptionally low LIV for all spheroid regions and was thus excluded from the statistical analysis as an outlier.

Figure 4 shows the morphological and mean D-OCT signal alterations for the HT-29 spheroid treated with SN-38. It was observed that the data in the TD-6 10-μM case had larger standard deviations (i.e., error bars in the figure) than the other cases for the mean LIV (Fig. 4b), LIV-based, and OCDS$_l$-based necrotic cell ratios (Fig. 4d,e, respectively). By observing the LIV images for the measured five spheroids in this case (Fig. 5), it was found that one spheroid case (spheroid-1) exhibited very low LIV values over the entire spheroid region. We suspect that this spheroid may be fragmented because of some cultivation issues or because of spheroid handling during the measurement. We, therefore, regard this case as an outlier and excluded it from the subsequent statistical analysis. The plots that include this outlier are presented with red spots and red lines, while those that exclude it are shown with orange color in Fig. 4.

The control spheroid volume (Fig. 4a) increased from TD-1 to TD-3, and then decreased slightly from TD-3 to TD-6. This volume alteration of the control spheroid may indicate the growth and spontaneous corruption of the HT-29 spheroid over the cultivation time. In contrast, the spheroids that were treated with SN-38 showed monotonic reductions in their spheroid volumes over time for all drug concentrations.

The mean LIVs (Fig. 4b) increased from TD-1 to TD-3 for all drug concentrations, and then decreased from TD-3 to TD-6 for the control sample (*P* = 0.006, Mann-Whitney test; supplementary Table S2).

The mean OCDS$_l$ in the 1-μM and 10-μM SN-38 concentration cases demonstrated significant reductions over time, as illustrated in (Fig. 4c). The significant reduction in the 1-μM case was found from TD-3 to TD-6 (P = 0.006, Mann-Whitney test), while that in the 10-μM was found from TD-1 to TD-3 (*P* = 0.018). For all other SN-38 concentrations, the treatment-time dependency was not observed. All statistical test results are presented in supplementary Table S2.

The LIV-based necrotic cell ratios demonstrated a reduction in the necrotic cell ratio from TD-1 to TD-3 for all SN-38 concentrations, and the ratio then became stable from TD-3 to TD-6. The reduction in LIV-based necrotic cell ratio from TD-1 to TD-3 was significant in the 1-μM and 10-μM cases of SN-38 (*P*= 0.047 and 0.010, respectively; Mann-Whitney test).

On the other hand, the OCDS$_l$-based necrotic cell ratios remain stable over time for low concentrations (i.e., the control and 0.1-μM), while that of the 10-μM case increases from TD-1 to TD-3 (*P* = 0.006). The ratio of the 1-μM shows a significant increase from TD-3 to TD-6 (*P* = 0.006). The results of this test are presented in Table S2 (supplementary).

The morphological and dynamic-OCT signal alterations of the HT-29 spheroid treated with SN-38 may be related to the drug mechanism of SN-38, as discussed later in the Discussion section. The OCT intensity, cross-sectional LIV and OCDS$_l$ results for the same data shown in Fig. 3 are presented in Figs. S4 and S5 in the supplementary material. In addition, the responses of one additional HT-29 spheroid under each treatment condition are presented as supplementary Fig. S6.

## Discussion

In the following seven paragraphs, we will summarize the characteristic OCT and D-OCT appearances of the MCF-7 spheroid results and interpret them based on the the drug action mechanism of PTX. At first, D-OCT imaging of the control MCF-7 spheroid [Fig. 1 (first row)] showed circular low dynamic signals (LIV and OCDS$_l$) surrounded by a high-dynamics shell. Similar image appearances were observed in our previous studies^31,39^. The low and high dynamics observed at the spheroid core and the periphery are believed to highlight the well-known necrotic core (caused by the lack of both oxygen and nutrient supply) and the viable rim of the tumor spheroids, respectively^3,52^. In addition, the increase of low dynamics area over the cultivation time [Fig. 1 (first row)] may indicate an increase in the necrotic area over the long cultivation time.

Similarity between the dynamics patterns of the MCF-7 spheroids treated with PTX for one day and that of the control spheroid was observed in Fig. 1 (first to third rows). This may indicate either the low efficacy of PTX or the resistance of the tumor cells when the treatment time is only one day.

Not only the specific findings mentioned above, the general tendency of the structural and D-OCT observed in this study can be explained as follows using the mechanism of PTX. Microtubules (MTs) are polymers that are formed by the polymerization of $\alpha$ and $\beta$ tubulin dimers^42^. MTs have three important functions in eukaryotic cells. First, MTs are the main constituents of the cytoskeleton, which maintains the cell structure and supports the cell with mechanical resistance to deformation^53^. Second, the MTs are involved in mitosis as the main constituents of the mitotic spindles^42,43^. Third, the MTs act as platforms for intracellular transport of the vesicles and organelles^42,54^.

For the functions described above, the MTs exhibit highly dynamic behavior. They exert rapid and stochastic phase transitions from extension (by polymerization) to shrinkage (by de-polymerization)^42,55^. MTs were found to grow at an average rate of 0.2–0.4 μm/s and shrink faster than this rate in most of the cells^56^. This dynamic instability allows the MTs tips to search for and bind to intracellular components, e.g., capturing chromosomes during mitosis^42,55^.

PTX is an MTs-stabilizing anti-cancer drug. It prevents MTs depolymerization and suppresses its dynamic behavior^40,41^. Therefore, it leads to cell cycle and mitotic arrest, which ultimately leads to cell death^40,41^.

The structural OCT findings for the PTX-treated MCF-7 spheroids showed spheroid shape corruption and volume reduction over the treatment time. This spheroid shape corruption may indicate malfunctioning of the first function of the MTs (i.e., maintaining the cell structure and the resistance to cell deformation) under the application of PTX. On the other hand, the reduction of the spheroid volume may indicate inhibition of mitotic cell divisions (the second function of the MTs) under the effect of PTX.

Furthermore, the spheroids treated with PTX showed reductions in their LIV and OCDS$_l$ signals over the treatment time. The LIV and OCDS$_l$ reductions may indicate a reduction of the intracellular motility/transport through the MTs (the third function of MTs) and may also indicate PTX-induced cell death. The growth rate of the MTs is 0.2–0.4 μm/s^56^, while the total acquisition time window of our method is 6.348 s. So, the MT may grow a few micrometers within the acquisition time. Although this is smaller than the resolution of OCT, it is large enough to decorrelate the OCT speckle pattern. And hence, LIV and OCDS$_l$ can be sensitive to the MT dynamics, and the reductions of LIV and OCDS$_l$ signals can be explained by the PTX-induced suppression of the MT dynamics and also by the reduction of intracellular transport through MTs (the third function of MTs).

Similar to the case of MCF-7, the OCT and D-OCT appearances of HT-29 spheroids can be explained based on the action mechanism of SN-38 as discussed in the following three paragraphs. The dynamics patterns of the TD-3 of HT-29 spheroid under all the SN-38 concentrations, except for the control case (Fig. 3) showed a clear low OCDS$_l$ layer at the periphery and this layer corresponded well with the peripheral dead cells (red fluorescence) observed in the fluorescence images. Among the three concentrations, the 10-μM case showed evident changes in the mean LIV and the mean OCDS$_l$ from TD-1 to TD-3 (Fig. 4b, c), and also showed the smallest spheroid volume among all concentrations, even at TD-1 (Fig. 4a). These results indicate that OCT and D-OCT imaging can demonstrate higher drug effects of 10-μM SN-38 than the other lower concentrations.

On TD-6, all the SN-38-treated spheroids at all concentrations (0.1, 1.0, and 10-μM) showed severe structural corruption. In comparison, on TD-3, these shape corruptions were not evident for all concentrations. However, the OCDS$_l$ images showed clear low OCDS$_l$ layers at the periphery that were not found in the control case. In addition, spheroid volume analysis (Fig. 4a) showed evidently smaller spheroid volumes, even at TD-3. These findings suggest that D-OCT can assess the more moderate drug effect of SN-38 than that can be visualized using non-D-OCT.

The alterations of OCT and D-OCT signals and the images of the HT-29 spheroids (Figs.  3, 4, respectively) can be understood more by taking the mechanism of SN-38 into account. Irinotecan is a DNA Topo-I inhibitor that is used to treat the advanced stages of colorectal cancer and it is also approved for second-line treatment in metastatic colorectal cancer^48,49^. SN-38 is an active metabolite of irinotecan, which is 100- to 1000-fold more active when compared with irinotecan itself^48,57^. Because Topo-I is an enzyme involved in DNA transcription and replication, the inhibition of Topo-I caused by SN-38 induces DNA damage and a transient S-phase (the cell cycle in which DNA is replicated) arrest. SN-38 application then leads to irreversible breaks in the single-strand-DNA that are subsequently transformed into double-strand-DNA breaks. As a result of these double-strand-DNA breaks, a series of different apoptotic-related signaling pathways are activated. This then results in apoptotic cell death^48,50^. The damage of DNA and subsequent DNA replication may suppress cell division, and this can account for the smaller spheroid size at TD-3. This DNA damage then progresses into apoptosis, and this can lead to the structural corruption observed at TD-6. The thin low-OCDS$_l$ (red) peripheral layers observed in all SN-38 treated spheroids on TD-3 may indicate the onset of apoptosis at the spheroid-drug interface. We suspect that the low-OCDS$_l$ may indicate the cell death process because the necrosis at the control spheroid center, which is another type of cell death, also exhibits low OCDS$_l$.

Both for the MCF-7 and HT-29 cases, LIV and OCDS$_l$ revealed different cellular processes, and hence, they are complement to each other. LIV is a measure of the magnitude of the temporal fluctuations in the dB-scaled OCT intensity and it may be sensitive to the magnitude of the intracellular motility. On the other hand, the OCDS$_l$ quantifies the speed of the OCT intensity fluctuations and it may be sensitive to the speed of the intracellular motility, as discussed elsewhere^31,39^. Because of this fundamental difference between them, the LIV and OCDS$_l$ may highlight different cellular processes. And this may explain why different image patterns appeared in LIV and OCDS$_l$. For example, in the control MCF-7 spheroids (Fig. 1), OCDS$_l$ shows clearer boundaries between the central and peripheral regions than LIV. In addition, in some cases, OCDS$_l$ shows tessellated domain structures such as those shown in Fig. 1 (magnified images) that are not visible in the corresponding LIV images. Although the identification of the cellular process that accounts for these LIV and OCDS$_l$ findings remains an open issue, we conclude that LIV and OCDS$_l$ provide different information and thus are complementary methods.

**Figure 6:**
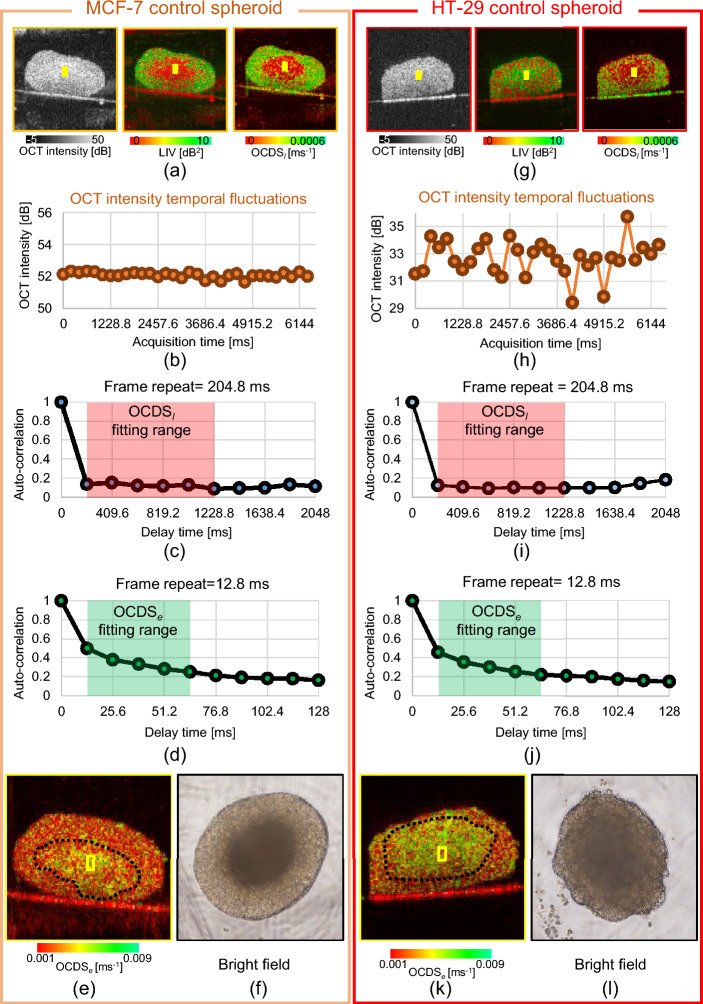
Detailed analysis of apparent pattern differences between the control MCF-7 (left) and HT-29 (right) spheroids. (**a**), (**g**) Cross-sections of the OCT intensity, LIV, and OCDS$_l$. (**b**), (**h**) Time course of the OCT intensity in the core areas of the spheroids (as indicated by the yellow boxes). MCF-7 shows very low fluctuations, while HT-29 shows high levels of fluctuation. (**c**), (**i**) Auto-correlation curves for both spheroids, showing rapid decay as the auto-correlation became very low at the first sampling point of the delay time (204.8 ms). (**d**), (**j**) The autocorrelation curves with high temporal density show details of the autocorrelation decay at short correlation delay time. (**e**), (**k**) OCDS$_e$ cross-sections of the same spheroids, showing the fast decay (green) at the center parts of the spheroid. These OCDS$_e$ images were computed from a cross-sectional frame sequence of 350 frames acquired at a single location. (**f**), (**l**) Bright-field microscopy images of the spheroids, which show the necrotic cores as dark central region.

The combination of OCT, LIV, and OCDS$_l$ can be useful to distinguish the cell types and drug-cell interaction types. In the OCT intensity images, no clear difference was found between the control spheroids of MCF-7 and HT-29 as shown in Fig. 6a,f. The OCDS$_l$ images also show the similar appearances for MCF-7 and HT-29. Namely, the center region shows low OCDS$_l$, while the periphery exhibits high OCDS$_l$. In contrast, the LIV shows opposite spatial patterns for these spheroids. Namely, the MCF-7 spheroid shows low and high LIV at its central and peripheral regions, respectively, while the HT-29 spheroid showed high and low LIV, respectively, in the corresponding regions (Fig. 6a, g).

To further investigate this difference, the time sequence of the OCT intensity (Fig. 6b, h) and the autocorrelation curves (Fig. 6c, i) are presented; these figures show the averaged intensity and the autocorrelation curves in the yellow boxed regions indicated in the OCT and D-OCT images. The OCT intensity fluctuation magnitude (Fig. 6b, h) is small for MCF-7 but it is large for the HT-29 spheroid. In addition, both autocorrelation curves (Fig. 6c, i) show that the correlation is strongly decayed even at the first computing point (204.8 ms), and thus the signal fluctuations in both cases are very fast. As the inter-frame separation time is 204.8 ms in our case, we suspect that the rapid decay of the autocorrelation may indicate fast biological activity (faster than the frame separation time) at the spheroid center. The rapid autocorrelation decay of OCT signal at the spheroid center was previously demonstrated by the authors and another research group, and it was suspected to indicate the activity of cell necrosis at the spheroid core^31,58^. For reference, the autocorrelation curves of slow decorrelation can be found in Fig. 2 of Ref. [^39^].

The current frame repeat time (204.8 ms) may enable the quantification of slow (second scale) tissue activity, such as the metabolic activity and the MTs dynamics of viable tumor cells at the spheroid periphery. These activities may explain the high OCDS$_l$ at the spheroid peripheries (Fig.6a, g). On the other hand, some fast processes, such as the necrotic activity at the spheroid center cannot be directly quantified by the OCDS$_l$ although it gives low OCDS$_l$ (red) (Fig. 6a, g). It is because the Nyquist frequency (2.44 Hz) determined by the current frame rate might be lower than the frequency components of the fast activity. Namely, higher frequencies than 2.44 Hz will suffer from frequency aliasing and appear as low frequencies. To quantify the fast activity and provide further validation of the fast autocorrelation decay at the spheroid center, a single B-scan location in the same spheroids was scanned 350 times in 4.48 s, i.e., it was scanned with much higher temporal density. This is the protocol that we presented for our original 2D dynamic imaging, and it enables yet another D-OCT contrast, the fast/early correlation decay speed (OCDS$_e$)^31^. This contrast is similar to OCDS$_l$, but the slope of the correlation decay is defined at an earlier time range [12.8, 64 ms] than OCDS$_l$, and thus it is more directly sensitive to the fast signal fluctuations. Figure 6d,j show the auto-correlation decay curves at the spheroid center computed from the data with the higher temporal density. These plots show the details of the autocorrelation decay at short correlation delay time. By fitting the slope of these curves at the time range of [12.8, 64 ms], we obtained the OCDS$_e$ images presented in Fig. 6e,k. As shown in these images, the OCDS$_e$ is high at the center regions of both spheroids. This further proves that the dynamics at the center regions are fast.

It is known that the center part of the spheroid becomes necrotic because of the lack of oxygen and other nutrients^6,52,59,60^. Necrosis is known to be completely progressed after 12–24 h of the start of the cell death process^61^. The necrotic cell death process includes a variety of processes. The most common one is the loss of plasma membrane integrity. The loss of plasma then leads to flow of cytoplasmic contents into the extracellular space^62^. Although the exact time scale of such cytoplasmic constituents’ flow is not defined, it is plausible to think that our frame repeat time (204.8 ms) and acquisition time window (6.348 s) can allow us to capture such activity as fluctuations of the OCT signal over the acquisition time. As a result, we suspect that the center regions of both spheroids are in the necrotic process. The difference in the magnitude of the signal fluctuations may be accounted by the time after the onset of cell death. Namely, we can assume that the onset of cell death for this particular MCF-7 spheroid was early, and hence the necrotic process has progressed, which results in the low fluctuation magnitude. On the other hand, the cell death onset of the particular HT-29 spheroid may be late, which means that the signal fluctuation magnitude is still large.

To further investigate these differences in the spheroid core activities, bright field microscopic images acquired using (IX71, Olympus) microscope with an objective with 4x magnification and an NA of 0.13 from the same samples are shown in Fig. 6f,l.

The bright field image of the MCF-7 spheroid (Fig. 6f) shows a dark appearance at its center with very clear boundaries surrounded by a bright appearance. The dark appearances at the spheroid center may indicate high cellular density, which may indicate the well-known irreversible nuclear condensation during cell necrosis^61^. In contrast, the HT-29 spheroid (Fig. 6l) shows a diffusive border. This may indicate that the necrotic core has not completely formed yet. These bright field images thus support our hypothesis.

In addition, our previous study involving time-lapse imaging of the MCF-7 spheroid demonstrated that the LIV at the central region was high at the early time points and it gradually degraded over hours^31^. This progressive reduction of LIV can be consistent with our hypothesis; the necrotic process progresses at the center region of the spheroid.

In addition to LIV, OCDS$_l$, and OCDS$_e$, several other D-OCT methods have been demonstrated. The similarities and differences among these D-OCT methods are summarized as follow. At first, these methods can be classified into two categories: magnitude evaluations and speed evaluations of the OCT signal fluctuations. The latter category can be categorized further into two subcategories that include autocorrelation analysis and time spectrum analysis.

The magnitude analysis may include the following research works. The cumulative sum (cumsum) method demonstrated by Scholler quantifies the signal fluctuation magnitude using a Brownian bridge model, and it has a high tolerance to bulk sample motion^63^. The motility amplitude method demonstrated by Oldenberg is also a metric to quantify the signal fluctuation magnitude^64^. These modalities are expected to visualize the fluctuation magnitudes of tissue and cellular dynamics.

The correlation analyses can quantify the speeds of the tissue dynamics. Leroux *et al.* computed the autocorrelation decay of an OCT signal and fitted it with a bi-exponential function, so that cellular dynamics with multiple speeds were quantified^65^.

Spectrum analysis is another method that can be used to quantify the temporal characteristics of the OCT signal fluctuations and tissue dynamics. The spectroscopic analysis demonstrated by Oldenburg *et al.* is one of the earliest demonstrations of this approach^64^. Apelian et al. combined a signal frequency analysis with a high-resolution full-field OCT imaging, and enabled high-resolution and functional imaging of ex vivo animal tissues^26^. Scholler et al. visualized the activity of retinal organoids through power-spectrum analysis of time-sequential OCT signals^28^. Recently, Münter et al. used a time-frequency analysis to enable three-dimensional visualization of tissue dynamics, and visualized the cellular scale dynamics of ex vivo animal tissues^29,34^. Both the correlation and spectral analysis methods may contrast the speed of the tissue dynamics.

In the present study, we used two methods: LIV and OCDS$_l$. LIV is a method to quantify the magnitude of the signal fluctuation, while OCDS$_l$ quantifies the speed of the fluctuation. As discussed in the combined interpretation of LIV and OCDS$_l$, the combination of these two methods provides a comprehensive interpretation of the dynamic processes occurring in the spheroids. However, the relationship between these D-OCT signal properties and the various types of intracellular motility is still not clear. The intracellular motilities include the transport of organelles and vesicles by molecular motors through microtubules, actin filaments, and other cytoskeletal structures^66,67^. In addition, thermal Brownian motion can cause the movement of cytoplasm and diffusive transportation of small molecules throughout the cell^68^. As a next step, we plan to construct a numerical simulation framework, which assumes arbitrary intracellular motions, and computes OCT and D-OCT signals. This simulator may help interrelate the D-OCT signals and the intracellular motilities.

In this and following two paragraphs, we discuss the resolution of D-OCT and its impact on detecting cellular dynamics. At first, the resolutions of our proposed D-OCT system, which are 14-μm (axial) and 18.1-μm (lateral), are lower than the size of a single cell. Hence, we cannot visualize the single cell structure. However, D-OCT can contrast tiny motions smaller than the resolution. In general, the OCT image exhibits small granular patterns (i.e., speckles), and the speckle pattern is altered by sub-wavelength motion of the sample. In the case of the present study, the motion of intracellular organelles alters the speckle pattern. And hence, the D-OCT can contrast the intracellular motions even those are smaller than the optical resolution. However, it should be noted that the detailed structure of dynamic domains in the tissue or cells cannot be resolved by D-OCT if the domain is smaller than the optical resolution. Namely, for a cell which is smaller than the resolution and exhibits intracellular motility, the motility in the cell can highlight the cell in the D-OCT image, but the shape of the cell, or equivalently, the shape of the dynamic domain, cannot be clearly delineated.

In the present study, we used two-types of cell-lines including MCF-7 with size of approximately 16.5 μm^69^ and HT-29 with size of 16.6 μm^70^, which are around or smaller than the optical resolution of our system. And hence, we cannot resolve the dynamic of each individual cell, but we still can contrast the cellular dynamics by D-OCT.

Our preliminary numerical studies suggest that LIV is highly affected by the wavelength of the probe beam, while it is less affected by the optical resolution^71,72^. In addition, the LIV seems to be sensitive to the ratio of static and dynamic scatterers in a resolution volume^73^. However, these studies are still preliminary, and more detailed studies are our ongoing work.

Using our proposed technique, we were able to acquire the 3D raw OCT data of each spheroid in 52.4 s. So, to measure the 96 spheroids in a 96-well plate, we need approximately 96 minutes. Although this measurement time is reasonable, the post-processing is a bit time-consuming. The reconstruction of the OCT intensity volume consisting of 4,096 frames captured at 128 B-scan locations takes around 30 min, whereas the LIV and OCDS$_l$ volume reconstructions take approximately 2 min and 15 min, respectively. So, the entire process to obtain the volumetric LIV and OCDS$_l$ of a spheroid takes about 47 min. However, it should be noted that our current OCT intensity reconstruction process includes high-accuracy phase-stabilization computation, which is used for the sake of future development of new D-OCT algorithms and is not really necessary for the LIV and OCDS$_l$ computations. The elimination of the phase stabilization process and future introduction of graphic-processing-unit- (GPU-) based processing may dramatically shorten the OCT intensity reconstruction time into ten-few seconds or a few seconds.

In addition to the points discussed above, we discuss the future topics of D-OCT in the supplementary material.

## Conclusion

The spatial patterns of the anti-cancer drug responses of two types of human tumor spheroid, including those derived from breast (MCF-7) and colon (HT-29) cancer cell-lines have been visualized using two types of D-OCT algorithms. The response patterns observed in D-OCT images were consistent with the corresponding fluorescence microscopy patterns. In addition, the spheroid volume and mean D-OCT signal values were computed. The time-course changes in these values revealed different trends among the two types of spheroid and anti-cancer drugs.

In conclusion, D-OCT can be used to highlight the difference in drug response patterns among different tumor spheroids and drug types. Therefore, D-OCT can be a useful tool for anti-cancer drug testing and the optimal selection of anti-cancer drugs.

## Methods

### Protocols and samples of drug response studies

**Figure 7:**
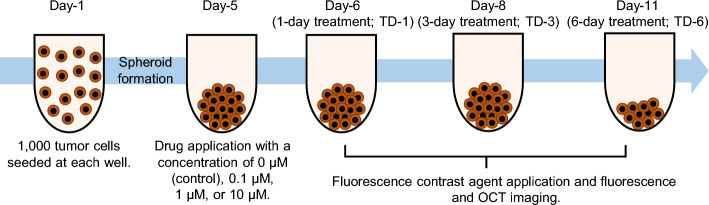
Time protocol for spheroid cultivation and measurement. The same time protocols were used for both MCF-7 and HT-29. 60 spheroids were cultured for both cell types. Each spheroid was seeded with 1000 cells on day-1. The drugs, i.e., PTX for MCF-7 and SN-38 for HT-29, were applied on day-5. On days-6, 8, and 11, fluorescence and then OCT imaging were performed. The fluorescence contrast agents were applied 3 hours before fluorescence imaging.

The tumor spheroid drug response evaluation study involves the use of two different human-derived tumor cell-lines, including human breast adenocarcinoma (MCF-7 cell-line) purchased from the Japanese Collection of Research Bioresources (JCRB) cell bank and human colon cancer (HT-29 cell-line) purchased from the American Type Culture Collection (ATCC). Both cell-lines were cultured using the same protocol in line with the study protocol depicted in Fig. 7. On day-1, 1000 tumor cells were seeded in each well of a ultra-low attachment 96-well-plate and 60 wells were prepared. The cells were cultured at a temperature of 37 $^\circ$C and the cell-culture chamber was supported with 5% CO$_2$. The culture medium contained a 1:1 mixture of Eagle’s minimal essential medium (EMEM; for MCF-7) or Dulbecco’s modified eagle medium (DMEM; for HT-29) and F12 (Invitrogen, Waltham, MA) with a 2% B-27 supplement (Invitrogen), 2 ng/mL of basic fibroblast growth factor (bFGF; Wako, Osaka, Japan), 2 ng/mL of epidermal growth factor (EGF; Sigma-Aldrich, St. Louis, MO), 100 U/mL of penicillin G, and 0.1 mg/mL of streptomycin sulfate (Wako, Osaka, Japan). The cells were aggregated with each other and formed one spheroid in each well on day-5. On the same day, each spheroid was then treated using 0.1 μM, 1 μM, or 10 μM of the relevant anti-cancer drugs. The MCF-7 spheroids were treated using paclitaxel (PTX, Sigma-Aldrich; also known as Taxol), while the HT-29 spheroids were treated using an active metabolite of irinotecan (SN-38, Chemscene LLC, NJ). In addition, untreated (0 μM) spheroids were retained as control samples. After drug application the spheroids were kept within the same culture environment.

The spheroids were then extracted from the cultivation and measured using the fluorescence and D-OCT microscopes at three time-points of days-6, 8, and 11, i.e., 1-, 3-, and 6-days after drug application, respectively. Here, we also denote the days 6, 8, and 11 as the 1st, 3rd, and 6th days of treatment (TD-1, -3, and -6). At each time-point, five spheroids were measured for each of the four drug concentrations, including control (0 μM), meaning that 20 spheroids were measured in total. It should be noted that, at each treatment condition different spheroid is measured. Namely, the spheroids are not the same individuals either over treatment time or along the drug concentrations. The measurements were performed in room temperature (25 $^\circ$C).

### Fluorescence microscopic imaging

As a gold standard reference measurement of the spheroids viability, live/dead fluorescence assays were performed using the THUNDER imager DMi8 (Leica Microsystems, Wetzlar, Germany) with a microscopic objective lens featuring a numerical aperture (NA) of 0.12. Two contrast agents were applied for three hours before the fluorescence imaging. The first agent is calcein acetoxymethyl (Calcein-AM; Dojindo, Kumamoto, Japan), which stains the living (viable) cells and emits a green fluorescence signal. The second agent is propidium iodide (PI; Dojindo), which contrasts the dead cells by emitting red fluorescence signal.

### 3D D-OCT imaging

#### OCT device

A polarization-sensitive Jones matrix swept-source OCT (JM-OCT) was used to perform the three-dimensional (3D) tissue dynamics imaging. A light source with a central wavelength of 1.3 μm and a scanning speed of 50,000 A-lines/s was used. The axial (in tissue) and lateral resolutions were 14 μm and 18.1 μm, respectively. The complete specification for the JM-OCT has been published elsewhere^51^. Although this device is polarization-sensitive, only polarization-insensitive OCT images, which are the average of four OCT intensity images acquired through four polarization channels, were used for the tissue dynamics imaging. Here we avoided using polarization-sensitive information to make our D-OCT method compatible with standard non-polarization-sensitive OCT devices.

#### OCT data acquisition protocol

The spheroid samples were transferred to our 3D JM-OCT system after fluorescence imaging. For the 3D dynamics imaging, the lateral imaging field was divided into eight regions. Each region consists of 16 B-scan locations and was scanned repeatedly using a quick raster scanning protocol for 32 times. The frame repetition time is 204.8 ms, while the time separation between the first and last frame(time window) is 6.348 s. The OCT volume consists of a total of 128 locations and was captured in 52.4 s. The scanning volume was 1 $\times$ 1 $\times$ 2.91 mm$^3$ for the fast scan lateral $\times$ slow scan lateral $\times$ depth directions. Further details about the 3D dynamics scanning protocol are available elsewhere in the literature^39^.

It should be noted that the fluorescence image and D-OCT images of the same spheroid may not be at exactly the same location of the sample. It is because the fluorescence and *en face* OCT imaging were performed in different laboratories, and hence the spheroid position may be altered during the laboratory-to-laboratory transportation. So, in some cases presented in the Results section, the fluorescence and dynamics OCT images show slightly different patterns.

#### D-OCT algorithm: Logarithmic intensity variance (LIV)

**Figure 8:**
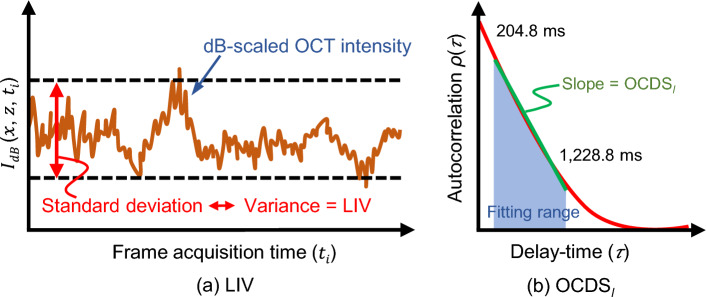
Schematic depictions of two D-OCT algorithms:(**a**) LIV and (**b**) OCDS$_l$. LIV is defined as the temporal fluctuation (variance) of the dB-scaled OCT intensity. As a result, LIV is expected to be sensitive to the magnitude of the tissue dynamics. On the other hand, OCDS$_l$ represents the speed of the dynamics and is defined as the decorrelation rate, i.e., as the slope of the auto-correlation curve [red curve in (**b**)] of the dB-scaled OCT intensity. The slope is computed here by performing a linear regression of the auto-correlation curve over a specified delay-time range [204.8, 1,228.8 ms].

The time-sequential OCT signals were processed using logarithmic-intensity-variance (LIV) algorithm^31^. The LIV is a measure of the magnitude of the temporal fluctuations of the dB-scaled OCT signals at each location in the tissue as illustrated in Fig. 8a, and it is defined as:

1 $$\begin{aligned} \textrm{LIV}(x,z) = \frac{1}{N} \sum _{i = 0}^{N-1} \left[ I_{dB}(x,z,t_i) - \left\langle {I_{dB}(x,z)}\right\rangle _{t} \right] ^2 \end{aligned}$$

where $I_{dB}(x,z,t_i)$ is the dB-scaled (logarithmic) OCT intensity, $t_i$ is the sampling time for the *i*-th frame, where *i* = 0, 1, 2,...., *N*-1, and *N* is the total number of frames, which is 32 in our case, and $\left\langle {}\right\rangle _{t}$ represents averaging over time.

Because the LIV is a measure of the magnitude of the signal fluctuations, it is believed to be sensitive to the magnitude of the intracellular motility.

An LIV image is presented as a pseudo-color image in which the hue and the value of the HSV (hue, saturation, value) color representation are set as the LIV and the averaged OCT intensity image, respectively. The color saturation is set to 1 (the maximum) for all the pixels.

#### D-OCT algorithm: late OCT correlation decay speed (OCDS$_l$)

The time-sequential OCT signals are also processed using yet another D-OCT algorithm: “late OCT correlation decay seed (OCDS$_l$).” OCDS$_l$ is defined as the speed (rate) of the auto-correlation decay of the sequentially captured dB-scaled OCT intensity and it is expected to be sensitive to the slow tissue dynamics^31^.

To obtain the OCDS$_l$, the temporal auto-correlation $\rho _A(\tau _i; x, z)$ was first computed as:

2 $$\begin{aligned} \rho _A(\tau _i; x, z) = \frac{\textrm{Cov}\left[ I_{dB}(x,z,t_i),\, I_{dB}(x,z,t_i+\tau _i)\right] }{\textrm{Var}\left[ I_{dB}(x,z,t_i)\right] \textrm{Var}\left[ I_{dB}(x,z,t_i+\tau _i)\right] }, \end{aligned}$$

where the numerator represents the covariance between $I_{dB}(x,z,t_i)$ and $I_{dB}(x,z,t_i+\tau _i)$, and $\textrm{Var}[\,\,]$ represents the variance. $\tau _i$ represents the delay time, which is defined as $i \Delta t$ where *i* is an integer variable. $\Delta t$ is the B-scan repetition time, which is 204.8 ms in this case.

The OCDS$_l$ is then defined as the correlation decay rate, i.e., as the slope of $\rho _A(\tau _i; x, z)$ over a specific range of $\tau$ = [204.8, 1228.8 ms] as illustrated in Fig. 8b.

An OCDS$_l$ image is presented as a pseudo-color image, which is generated in the same manner as the LIV image, but in this case the hue is the OCDS$_l$ signal. Further details about the LIV and OCDS$_l$ algorithms are published elsewhere^39^.

#### OCT-based volumetric quantification

To quantify the volumetric alterations of the morphology and viability of the spheroid cells, the spheroid volume was computed via B-scan by B-scan segmentation. The segmentation was performed using the find-connected-region plugin of the Fiji ImageJ software with a manually defined OCT intensity threshold. The initial segmentation contained the bottom surface of the well-plate because it shows high OCT intensity. This well-plate surface was removed manually.

In addition to the spheroid volume, the mean values of the LIV and OCDS$_l$ over the entire spheroid volume were computed using the segmentation results. Furthermore, by applying empirically defined cut-offs of 3 dB$^2$ and 2 $\times 10^{-4}$ ms$^{-1}$ for the LIV and OCDS$_l$, respectively, the necrotic cells volume (i.e., the volume of the LIV and OCDS$_l$ signals that are lower than the cut-offs in each case) is computed^39^. The necrotic cell ratio (= necrotic volume / entire spheroid volume) is then computed. These computed quantities are plotted as a function of the treatment time for each drug concentration, as shown in the Results section.

### Supplementary Information

Supplementary Information.
