# Supplementary material for: Label-free drug response evaluation of human derived tumor spheroids using three-dimensional dynamic optical coherence tomography
Source: Sci Rep. 2023 Sep 16;13:15377. doi: 10.1038/s41598-023-41846-3 (PMC10505213; doi:10.1038/s41598-023-41846-3)
Supplement: Supplementary file 1 — Supplementary Information. [file 41598_2023_41846_MOESM1_ESM.pdf]

Supplementary material

Label-free drug response evaluation of human derived tumor spheroids using three-dimensional dynamic optical coherence tomography

Ibrahim Abd El-Sadek[1,2], Larina Tzu-Wei Shen[3], Tomoko Mori[3], Shuichi Makita[1], Pradipta Mukherjee[1], Antonia Lichtenegger[1,4], Satoshi Matsusaka[3], and Yoshiaki Yasuno[1]

[1]*Computational Optics Group, University of Tsukuba, Tsukuba, Ibaraki 305-8573, Japan*

[2]*Department of Physics, Faculty of Science, Damietta University, New Damietta City, 34517, Damietta, Egypt*

[3]*Clinical Research and Regional Innovation, Faculty of Medicine, University of Tsukuba, Ibaraki 305-8575, Japan*

[4]*Center for Medical Physics and Biomedical Engineering, Medical University of Vienna, Wahringer Gurtel 18-20, 4L, 1090, Vienna, Austria.*

Abstract

Here we present the cross-sectional OCT intensity, en face OCT intensity, and cross-sectional LIV and $OCDS_l$ images of the spheroid pretested in the full-length manuscript. In addition, the measurement results of additional MCF-7 and HT-29 spheroids are provided and they are consistent with those shown in the full-length manuscript. Furthermore, the tables elaborating the statistical significance analysis are included.

Supplementary figures

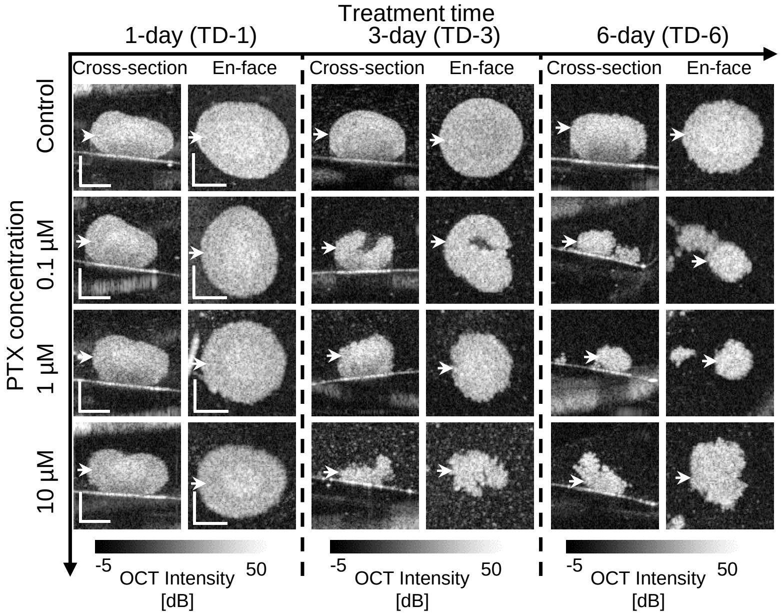

Figure S1: Cross-sectional and *en face* OCT intensity images of the MCF-7 spheroid presented in Fig. 1 in the full-length manuscript. The intensity images show the morphological alteration of the spheroid, while no tissue activity contrast can be observed. Scale bars represent 200 µm.

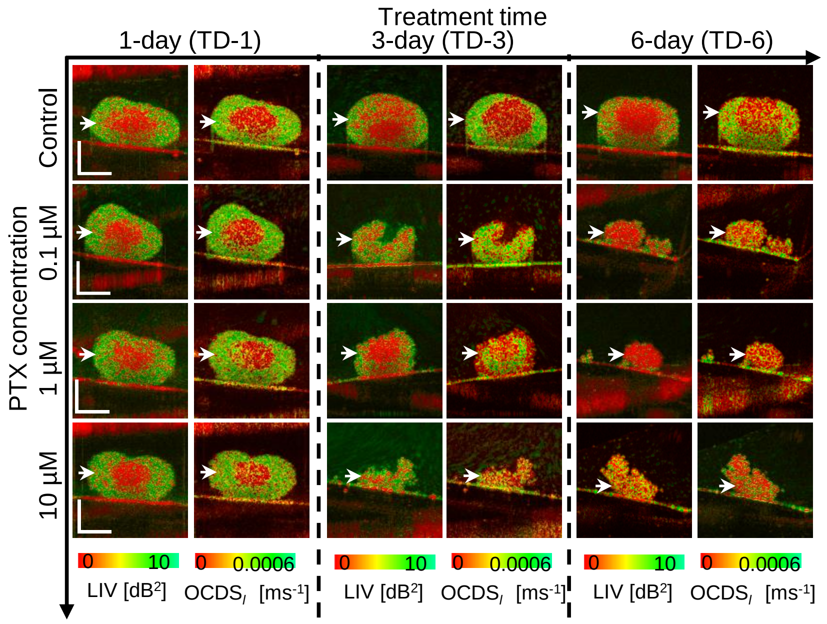

Figure S2: Cross-sectional LIV and OCDS$_l$ images of the MCF-7 spheroid treated with PTX. The images were extracted from the locations indicated by white the arrow heads on the *en face* images in Fig. 1 in the full-length manuscript, and they show similar tendency to the *en face* images. Scale bar represent 200 µm.

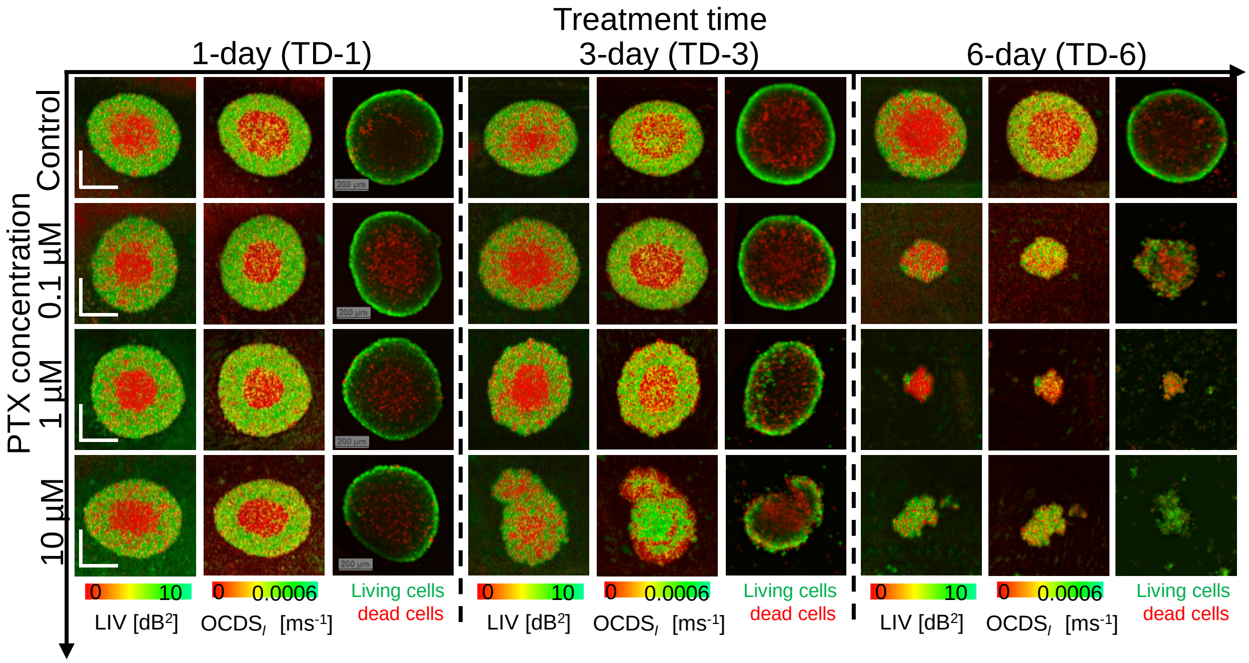

Figure S3: *En face* LIV and OCDSl images of additional MCF-7 spheroids treated with PTX. The measurement and treatment were performed with the same protocol as that of Fig. 1 in the full-length manuscript. The images are presented in the same order as Fig. 1 in the full-length manuscript. Scale bars are applicable for all the LIV and OCDS$_l$ images and they represent 200 µm.

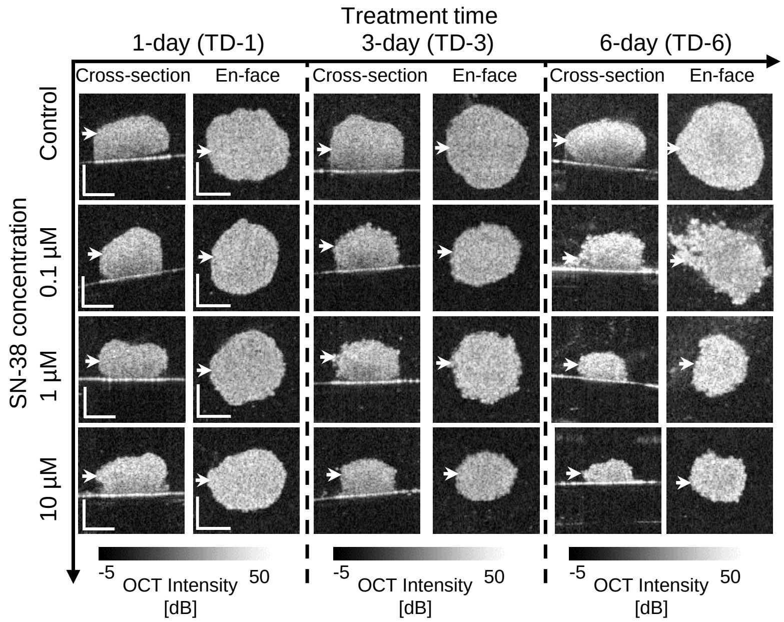

Figure S4: Cross-sectional and *en face* OCT intensity images of the HT-29 spheroids presented in Fig. 3 in the full-length manuscript. The intensity images show the morphological alteration of the HT-29 tumor spheroid. Scale bars represent 200 µm.

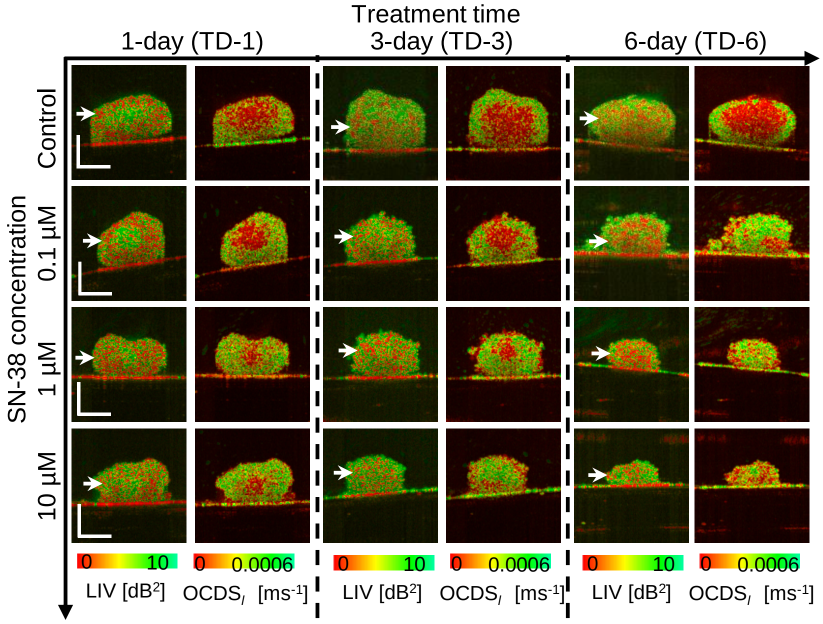

Figure S5: The cross-sectional LIV and OCDS$_l$ images of HT-29 spheroids extracted from the locations indicated by the white arrow heads in Fig. 3 in the full-length manuscript. The cross-section LIV and OCDS$_l$ images show similar appearances to those of the *en face* images presented in Fig. 3. Scale bars represent 200 µm.

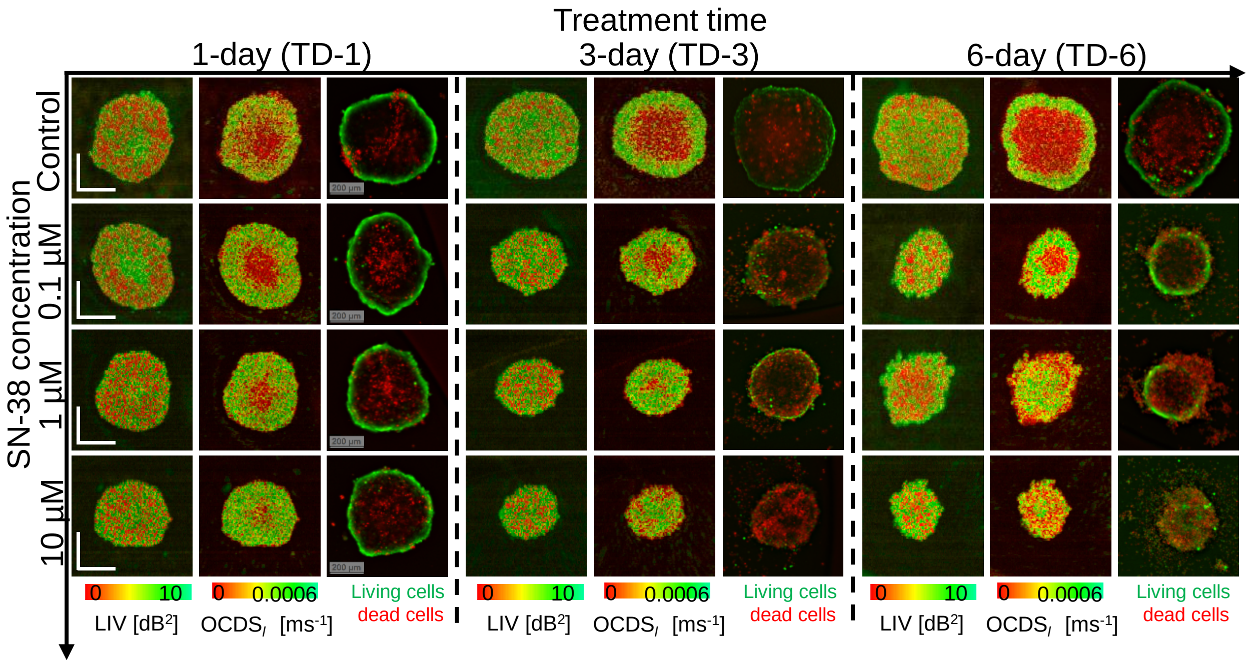

Figure S6: The *en face* LIV and OCDS$_l$ images of additional HT-29 spheroids treated with SN-38. The images are presented in the same manner as Fig. 3 in the full-length manuscript. The LIV and OCDS$_l$ images show similar patterns to those presented in Fig. 3 in the manuscript. Scale bars represent 200 µm.

Supplementary tables

Table S1: Mann-Whitney significance test results of MCF-7 spheroid response to PTX based on the mean LIV, mean OCDS$_l$, LIV cut-off based necrotic cells ratio, and OCDSl cut-off based necrotic cells ratio. The tests were performed between each pair of treatment time points. The * mark indicates the significant conditions (P < 0.05).

PTX concentration / Time point pairs	Control [U-value, P-value]	0.1-μM [U-value, P-value]	1-μM [U-value, P-value]	10-μM [U-value, P-value]
TD-1, TD-3 Mean LIV-based	[4.0, 0.047]*	[2.0, 0.018]*	[0.0, 0.006]*	[5.0, 0.071]
TD-3, TD-6 Mean LIV-based	[0.0, 0.006]*	[0.0, 0.006]*	[2.0, 0.018]*	[5.0, 0.071]
TD-1, TD-6 Mean LIV-based	[0.0, 0.006]*	[0.0, 0.006]*	[0.0, 0.006]*	[0.0, 0.006]*
TD-1, TD-3 Mean OCDS$_l$-based	[3.0, 0.030]*	[0.0, 0.006]*	[2.0, 0.018]*	[0.0, 0.006]*
TD-3, TD-6 Mean OCDS$_l$-based	[0.0, 0.006]*	[0.0, 0.006]*	[3.0, 0.030]*	[7.0, 0.148]
TD-1, TD-6 Mean OCDS$_l$-based	[0.0, 0.006]*	[0.0, 0.006]*	[0.0, 0.006]*	[0.0, 0.006]*
TD-1, TD-3 LIV-based necrotic cell ratio-based	[5.0, 0.071]	[3, 0.030]*	[0.0, 0.006]*	[4.0, 0.047]*
TD-3, TD-6 LIV-based necrotic cell ratio-based	[0.0, 0.006]*	[0.0, 0.006]*	[4.0, 0.047]*	[3, 0.030]*
TD-1, TD-6 LIV-based necrotic cell ratio-based	[0.0, 0.006]*	[0.0, 0.006]*	[0.0, 0.006]*	[0.0, 0.006]*
TD-1, TD-3 OCDS$_l$ -based necrotic cell ratio-based	[3.0, 0.030]*	[2.0, 0.018]*	[1.0, 0.010]*	[0.0, 0.006]*
TD-3, TD-6 OCDS$_l$ -based necrotic cell ratio-based	[0.0, 0.006]*	[0.0, 0.006]*	[3.0, 0.030]*	[5.0, 0.071]
TD-1, TD-6 OCDS$_l$ -based necrotic cell ratio-based	[0.0, 0.006]*	[0.0, 0.006]*	[0.0, 0.006]*	[0.0, 0.006]*

Table S2: Mann-Whitney significance test results of HT-29 spheroid response to SN-38 based on the mean LIV, mean $OCDS_l$, LIV cut-off based necrotic cells ratio, and OCDSl cut-off based necrotic cells ratio. The tests were performed between each pair of treatment time points. The * mark indicates the significant conditions (P < 0.05).

SN-38 concentration / Time point pairs	Control [U-value, P-value]	0.1-μM [U-value, P-value]	1-μM [U-value, P-value]	10-μM [U-value, P-value]
TD-1, TD-3 Mean LIV-based	[5.0, 0.071]	[7.0, 0.148]	[5.0, 0.071]	[1.0, 0.010]*
TD-3, TD-6 Mean LIV-based	[0.0, 0.006]*	[9.0, 0.265]	[8.0, 0.201]	[5.0, 0.135]
TD-1, TD-6 Mean LIV-based	[11.0, 0.417]	[11.0, 0.417]	[6.0, 0.105]	[3.0, 0.055]
TD-1, TD-3 Mean $OCDS_l$-based	[9.0, 0.265]	[6.0, 0.105]	[8.0, 0.201]	[2.0, 0.018]*
TD-3, TD-6 Mean $OCDS_l$-based	[6.0, 0.105]	[7.0, 0.148]	[0.0, 0.006]*	[3.0, 0.055]
TD-1, TD-6 Mean $OCDS_l$-based	[10.0, 0.338]	[10.0, 0.338]	[0.0, 0.006]*	[0.0, 0.009]*
TD-1, TD-3 LIV-based necrotic cell ratio-based	[5.0, 0.071]	[4.0, 0.047]*	[5.0, 0.071]	[1.0, 0.010]*
TD-3, TD-6 LIV-based necrotic cell ratio-based	[7.0, 0.148]	[10.0, 0.338]	[5.0, 0.0718]	[8.0, 0.356]
TD-1, TD-6 LIV-based necrotic cell ratio-based	[5.0, 0.0718]	[6.0, 0.105]	[4.0, 0.047]*	[1.0, 0.018]*
TD-1, TD-3 $OCDS_l$ -based necrotic cell ratio-based	[9.0, 0.265]	[10.0, 0.338]	[9.0, 0.265]	[0.0, 0.006]*
TD-3, TD-6 $OCDS_l$ -based necrotic cell ratio-based	[6.0, 0.105]	[7.0, 0.148]	[0.0, 0.006]*	[4.0, 0.088]
TD-1, TD-6 $OCDS_l$ -based necrotic cell ratio-based	[8.0, 0.201]	[10.0, 0.338]	[0.0, 0.006]*	[0.0, 0.009]*

Supplementary Discussion (Future topics of dynamic optical coherence tomography imaging for tumor spheroids)

In this section, we are going to discuss some possible future topics of our dynamic optical coherence tomography (D-OCT) method.

In this study, we used only one type of morphological metric, the spheroid volume, to assess the drug response of spheroids. For future studies we may consider more sophisticated morphological features, such as sphericity, convexity, solidity, diameter, and perimeter, which are known to be important metrics for the evaluation of the spheroid response to therapeutics[1].

In the current study design, we first performed fluorescence imaging with invasive fluorescence agents and then performed OCT measurements. This measurement order was chosen because of the physical locations of the two laboratories, one was for the spheroid cultivation and the fluorescence imaging, and the other was for OCT measurements. Although OCT is a non-invasive imaging modality, we have measured the spheroids with invasive agents in this protocol. In the future studies, it would be preferable to inverse the measurement order and perform the OCT imaging in a totally non-invasive manner.

In the current study design, the spheroids have been extracted from the cultivation environment for the OCT measurements. This extraction might harm the viability of the spheroids. For the future studies, it is important to integrate a cultivation chamber to the OCT device to enable on-site OCT imaging under cultivation environment.

Our dynamic OCT method provides two contrasts (LIV and $OCDS_l$) for the intracellular motility visualization of the tissue. The same raw OCT data can be used to estimate the scatterer density[2]. In addition, the polarization-sensitive OCT device used in this study can provide the polarization properties of the tissue, such as birefringence[3,4] and degree of polarization uniformity[5,6]. These multiple contrasts in addition to the D-OCT contrast may promote a more comprehensive understanding of the tissue properties. In addition, they can be used to synthesize new contrasts, for example, by using principal component analysis [7,8] and multi-contrast-based tissue segmentation[9], for specific applications. In future work, such approach may enable non-invasive virtual histopathology.

References

1. Aguilar Cosme, J. R., Gagui, D. C., Bryant, H. E. & Claeyssens, F. Morphological Response in Cancer Spheroids for Screening Photodynamic Therapy Parameters. *Frontiers in Molecular Biosciences* **8**, (2021).
2. Seesan, T. *et al.* Deep convolutional neural network-based scatterer density and resolution estimators in optical coherence tomography. *Biomed. Opt. Express, BOE* **13**, 168–183 (2022).
3. Sugiyama, S. *et al.* Birefringence imaging of posterior eye by multi-functional Jones matrix optical coherence tomography. *Biomed. Opt. Express, BOE* **6**, 4951–4974 (2015).
4. Li, E., Makita, S., Hong, Y.-J., Kasaragod, D. & Yasuno, Y. Three-dimensional multi-contrast imaging of in vivo human skin by Jones matrix optical coherence tomography. *Biomed. Opt. Express, BOE* **8**, 1290–1305 (2017).
5. Götzinger, E. *et al.* Retinal pigment epithelium segmentation by polarization sensitive optical coherence tomography. *Opt. Express, OE* **16**, 16410–16422 (2008).
6. Makita, S., Hong, Y.-J., Miura, M. & Yasuno, Y. Degree of polarization uniformity with high noise immunity using polarization-sensitive optical coherence tomography. *Opt. Lett., OL* **39**, 6783–6786 (2014).
7. Bazant-Hegemark, F. & Stone, N. Near real-time classification of optical coherence tomography data using principal components fed linear discriminant analysis. *JBO* **13**, 034002 (2008).
8. Fang, L. *et al.* Automatic classification of retinal three-dimensional optical coherence tomography images using principal component analysis network with composite kernels. *JBO* **22**, 116011 (2017).
9. Hsu, D. *et al.* Quantitative multi-contrast in vivo mouse imaging with polarization diversity optical coherence tomography and angiography. *Biomed. Opt. Express, BOE* **11**, 6945–6961 (2020).
